# Casts of the Uriniferous Tubules

**Published:** 1896-08

**Authors:** Thomas B. Carpenter

**Affiliations:** Buffalo, N. Y. 325 Jersey Street


### BUFFALO MEDICAL JOURNAL.

VOL. XXXVI. AUGUST, 1896. No. 1.

### Original Communications.

### CASTS OF THE URINIFEROUS TUBULES.

By THOMAS B. CARPENTER, M. D., Buffalo, N. Y.

TUBE CASTS, renal casts or cylinders are casts of the uriniferous tubules, caused by the presence of a coagulable substance in the tubule. This substance when coagulating entangles in its body anything that may have been surrounded by it while yet a liquid. From this fact, or from differences in composition of the coagulable substance, the various casts take their names. If loose epithelial cells happen to be in the tubule before solidification of this material we have an epithelial cast ; if blood, a blood cast, and so on for the other varieties.

Very great variations in size are met with, the diameter and length varying with the size and condition of the tubule in which formed. Casts from the convoluted tubules are much narrower than those from the straight or collecting tubules. If the tubules are denuded of their epithelial lining we get an increase in diameter corresponding to the thickness of the epithelial layer. In many cases great increase in diameter is due to dilatation of the tubule, due to plugging near its orifice and consequent packing of the cast-forming material above the plug. As a rule, the variation in diameter is from 1-2500 to 1-500 of an inch. The length may vary greatly, but we never see complete casts of the whole tubule, for they must necessarily become broken in their passage downward. Casts of the straight tubules are usually the longer.

Regarding the source and composition of the cast-forming material there is considerable difference of opinion. Some investigators consider the material to be a coagulable constituent of the blood introduced into the tubule by capillary rupture or otherwise. This is undoubtedly true in some cases. Others claim that the colorless casts do not consist of fibrin and differs materially from other varieties of albumin. Then again, some consider these colorless casts to be a product of a disturbance of nutrition of the glandular epithelium lining the tubules, or, in other words, a degenerative product. Others again think that the epithelial cells have the power to elaborate and pour into the tubule a coagulable substance not a product of degeneration. Since we can have casts formed in tubules entirely bereft of epithelial lining, it follows that the last two theories cannot be true in all cases. It is probable that all theories mentioned are correct in respective cases. Staining experiments are under way that may be helpful in clearing up some disputed points regarding the composition of certain casts.

In the following pages I shall endeavor to classify the various varieties of casts according to their characteristics, that they may be more available as aids to accurate diagnosis.

For all practical purposes we may divide casts into the following varieties: hyaline, fine granular, coarse granular, brown granular, epithelial, blood, fibrinous, fatty, waxy, crystalline, bacterial and purulent. They differ from each other either in composition of the coagulable material forming them or in the character of the substances which become entangled in their bodies when coagulating. In many instances sharp lines of demarcation cannot be made between certain varieties, but it is essential that this be done to the fullest extent, for in this way alone is it possible to derive information of the probable extent of inflammatory or degenerative processes in the kidneys.

Hyaline casts, so called from their structureless and hyaline appearance, come from tubules where the epithelial lining is intact, firmly attached to the basement membrane or from tubules entirely denuded of epithelium. They are structureless and colorless, very difficult to see in many cases, and their recognition is of the highest importance. As a rule, the cast is not entirely hyaline, but has a few granules adherent in different places or perhaps a few minute oil drops. The pure hyaline casts are difficult to see, being of about the same refractive index as the urine in which floating, and, since they are of about the same specific gravity, settle with difficulty. The threads of mucus, so-called mucous casts found in highly acid urines, may easily be mistaken for hyaline casts. They do not come from the kidneys and differ from hyaline casts by their usual great length, fibrillated appearance, difference in diameter at different places and terminating at one or both ends in a point. Hyaline casts are found in all affections of the kidneys, whether temporary or permanent.

It must be borne in mind that some observers claim the presence of hyaline casts in normal urines. An experience of ten years with many thousands of normal and pathological urines has failed to furnish such evidence to the writer.

Granular casts are nothing more than the hyaline variety rendered opaque by a mixture with or covering of granular matter derived from degenerated epithelium or blood. If from blood, we have the heavy brown granular cast. Whether a cast is finely or coarsely granular is of some importance. As a rule, the more acute the affection the more granular débris and the coarser the casts. The fine granular casts are found under about the same conditions as the hyaline variety. The coarse and brown granular casts are, as a rule, found only in acute processes, as severe active hyperemia and acute parenchymatous nephritis.

Epithelial casts are, as the name suggests, casts that are covered wholly or in part with renal epithelial cells. True epithelial cylinders are also found, composed of a shell of cells due to the whole epithelial lining coming away in one piece. They are rarely seen. The epithelial casts proper were primarily, at least in many cases, true hyaline casts, but, due to disease of the tubular lining cells, they became loosened or detached and affixed to the cast. Oftentimes we find the epithelium on one side only. They are recognised without difficulty and when present in any amount usually denote either an active hyperemia or acute parenchymatous nephritis.

Blood casts may be either hyaline casts originally, which entangled blood in their substance while coagulating, or they may be pure clotted blood introduced into the tubule by capillary rupture. It is usual to consider a cast as a blood cast even though it has comparatively few blood corpuscles on its surface. In size they are the same as other casts subjected to the same conditions. They are easily recognised, particularly if formed of clotted blood, and as a rule the corpuscles on the surface are very distinct. They are found only in acute processes, the typical specimens occurring in acute parenchymatous nephritis.

Fibrinous casts are those highly refractive casts varying in color from light yellow to deep yellow or even brown. Usually they are of a large diameter and have a peculiar convoluted appearance, as if the tubule in which formed was dilated or sacculated, or as if the substance was introduced under pressure. They are supposed to be formed by the exudation and coagulation of blood plasma. This variety is found in acute parenchymatous and septic nephritis.

Waxy casts, so called from their wax-like appearance, are colorless like the hyaline, but very refractive. Often they have the convoluted appearance of the fibrinous but without the color. The composition of these casts is not known. Many give the amyloid reaction with iodine solution, but this is not the rule except with those found in amyloid degeneration. They are found in all chronic diseases of the kidneys nearing a fatal termination, but, as would be supposed, their appearance in amyloid disease is comparatively early. In chronic interstitial and parenchymatous nephritis they appear late and always warrant the prognosis of a speedy fatal termination. Many writers make no distinction between the waxy and hyaline casts, and fibrinous also are indiscriminately called waxy, hyaline or refractive. The question of differentiation between these three varieties of casts is of considerable importance, both from a diagnostic and prognostic point of view. The fibrinous casts are highly refractive, always colored and are found only in acute processes. The waxy casts are similar in appearance to the fibrinous, but without color. They are found only in chronic affections. The hyaline casts are colorless but not refractive like the waxy.

Fatty casts are those containing oil drops on their surface, either free or as fatty degenerated cells. The basis substance is the same as the hyaline casts ; but during or after setting of the plastic substance the oil or fatty degenerated cells become entangled or adherent to its surface. They are found in chronic parenchymatous nephritis, in acute parenchymatous nephritis during convalescence and in severe long-continued active hyperemia.

Crystalline casts—that is, casts containing uric acid, urates, calcic oxalate or cystine—are occasionally met with. These crystals may accidentally become adherent to the cast after it has left the tubule, but casts are met with containing them imbedded in their substance and are interesting as showing that these crystals may separate in the kidney tubules. In infants suffering from uric acid infarction of the kidneys we find casts in the urine composed almost entirely of uric acid and urates.

Bacterial casts are those composed of or containing bacteria. Usually they are of large size and resemble a granular fibrinous cast.

As the bacteria are at rest they have simply a granular appearance, but proper staining and examination with a high power will reveal their true nature. They are associated with purulent casts and found only in septic nephritis.

Purulent casts are found under the same conditions as the preceding. In general appearance they may closely resemble epithelial casts, but as a rule they are of larger diameter ; coming from the straight tubules and their associates, free pus in quantity and bacterial casts render their nature evident. By the addition of acetic acid the granular matter of the corpuscles may be dissolved, thus showing up the two or three nuclei characteristic of pus.

An absolute diagnosis of a pathological condition in the kidneys cannot be made from casts alone. We must take into consideration the sediment as a whole, and even then in many cases we must have a chemical analysis of the urine and know the average amount passed in twenty-four hours. This last factor is of the greatest importance.

Active hyperemia is characterised by hyaline and fine granular casts, with an occasional blood and epithelial cast from the larger tubules. If very protracted and severe we may find a few fibrinous and brown granular casts and possibly a few fatty epithelial casts. In connection with these casts we find free blood and renal cells. The renal epithelial cells are usually of the larger variety, tending toward the battledore shape and come from the straight tubules. In mild cases this sediment may resemble that of amyloid degeneration or interstitial nephritis. It is differentiated from these two affections by the fact that the total amount of urine is always less than normal, while in amyloid and interstitial disease the amount is largely increased.

In passive hyperemia we rarely find more than a few hyaline and fine granular casts associated with a few renal cells and amorphous urates. This affection is even more likely to be confounded with amyloid or interstitial than the preceding. It differs from both by an increase in the total solids and having a small amount, high color and high specific gravity, although at times we may have a low specific gravity and pale color, but never so low as in amyloid or interstitial disease.

In acute parenchymatous nephritis, in the early stages, we find blood, epithelial, brown granular and fibrinous casts, rarely hyaline and fine granular casts. These various casts are associated with much blood, leucocytes and renal cells of all kinds, all colored brown by blood pigment. As the case progresses we begin to find fatty elements—fatty casts, fatty renal cells and compound granule cells. The appearance of these fatty elements in a case of acute nephritis indicates the beginning of convalescence and the patient will probably recover. As the fat increases the blood diminishes, as do also the blood, fibrinous and brown granular casts. Finally the fat disappears also, and we find only renal cells, hyaline and fine granular casts, with few blood globules and an occasional blood cast. The fatty stage of acute nephritis is very liable to be confounded with the chronic parenchymatous nephritis, therefore a guarded diagnosis should be given. The principal point of difference is the presence of more or less blood in the acute form.

In the active stage of chronic parenchymatous nephritis we find hyaline, granular, fatty and fatty epithelial casts, associated with free fat, fatty renal cells, compound granule cells and amorphous urates. During the inactive stage, when the patient feels better and has returned to work, the sediment decreases in amount, though still containing the same elements with the exception of the amorphous urates, which have entirely disappeared. If life is prolonged a sufficient length of time, which is rarely the case, the large fatty kidney becomes atrophied and we have the so-called atrophic stage. The sediment, and in fact all the characteristics of the urine in this stage are very similar to the interstitial nephritis. The only point of difference is the presence of fatty elements in the parenchymatous variety. If at any time during the course of the disease we find waxy casts we may soon expect a fatal termination.

In amyloid degeneration we find hyaline, fine granular and at a late period waxy casts. The waxy casts appear much earlier in this affection and do not have the same importance as when appearing in interstitial or parenchymatous nephritis.

In chronic interstitial nephritis we find a few hyaline and fine granular casts associated with a few renal cells. This form of Bright's disease is the most common, most important and the most difficult to detect. As a rule the disease continues for years with slight symptoms, and in many cases no symptoms at all, until uremic intoxication appears. After this, if the patient lives, we find waxy casts in addition to the first stage elements. The characteristics in this affection and in amyloid degeneration are about the same. As a rule the amount is larger and the specific gravity higher in amy loid disease. The solids are much diminished in the interstitial, while in the amyloid they are normal or only slightly diminished

The amount of albumen throughout the whole course of interstitial is very small and may be absent for a few days at a time. It never exceeds 0.25 per cent. In amyloid disease the amount of albumen is small at first, but gradually increases to 0.50 per cent. near the end.

It must be understood that this scheme of differential diagnosis from the sediment characteristics of the urine, is based on typical cases and is liable to considerable variation from complications of one pathological process with another. We may have many first stages in the course of an acute nephritis due to exposure or other causes, particularly in hospital cases. This, of course, would modify to some extent the sequence of changes in the characteristics of the sediment as mentioned. The fatty changes may take place and the sediment begin to clear up, when suddenly the whole preceding process may be repeated. In chronic parenchymatous nephritis we may have many acute exacerbations from time to time, and at such time the sediment presents the characteristics of both the acute and chronic nephritis. This condition of affairs resembles exactly the fatty stage in acute nephritis and the differentiation must be based on the chemical analysis and previous history. Complications of chronic parenchymatous and amyloid disease are not rare and this double process is difficult to diagnose. The sediment possesses the same characteristics as in pure chronic parenchymatous, but usually the amount of urine is larger and the color is paler. Previous history of extensive suppuration or syphilis and evidence of amyloid disease in other organs will confirm the diagnosis.

The commonest complication is interstitial, with parenchymatous nephritis, usually spoken of as diffuse nephritis. There is usually no great difficulty in diagnosticating the complicated condition, and as a rule the degree of one or the other can be determined with a fair degree of accuracy. As the parenchymatous changes increase, so does the amount of albumin and fatty elements, and the total amount of urine and the specific gravity decrease. As the interstitial variety predominates the converse applies.

The above examples of complicated conditions will illustrate how the character of the urine and sediment may be modified. If the results of examination are inadequate for a diagnosis the fault will probably be due to lack of care in examination, regarding which a few words will not be out of place.

Centrifugation of a sample is, of course, a valuable and perhaps the best means of obtaining a sediment for examination. But few physicians possess an apparatus of this character ; therefore the following instructions will enable one to obtain the best results without a centrifuge.

The glasses used for the collection of specimens must be scrupulously clean, particularly if they were used for the same purpose before. One drop of a stale sample will speedily render a new one unfit for examination. If the conditions are such that we may expect rapid decomposition, as in warm weather or in samples containing much pus, it may be prevented by using perfectly clean tightly-stoppered bottles, into which has been introduced a little salicylic acid, chloral hydrate or a few drops of chloroform. By this means a sample can be kept several days in good condition. If possible we should always use a part of the whole amount passed in twenty-four hours. If this cannot be obtained use that passed in the afternoon. It should be allowed to settle in a tall glass with a rounded concave bottom and parallel sides. The conical glasses so often used allow too much sediment to settle on the sides, where in this shape of glass we can usually find more casts than at the bottom. As a rule from four to eight hours is sufficiently long for settling, except in those cases where we have a pale urine, of low specific gravity, large amount and no appreciable sediment. In such cases—that is, of suspected interstitial nephritis—the sediment may contain only a few pure hyaline casts, associated with a few renal cells, and as a rule is small in amount, hard to find and settles with difficulty. In these cases allow it to settle for twenty-four hours. The use of staining fluids is rather superfluous, except perhaps in such cases as the last mentioned. A few drops of a concentrated solution of fuchsine or methylene blue may be added to the whole amount set aside to settle.

In those samples containing a heavy sediment of amorphous urates it is well to add about an equal bulk of warm water before setting aside to settle. This will accomplish their complete solution and leave the field clearer for careful observation. For the same reason large deposits of phosphates had better be dissolved. This can be accomplished by rendering the sample distinctly acid with acetic acid.

A great deal of the success in examining urinary sediments depends on the manner of using the pipette. The tip of the pipette should be drawn out long and at the orifice should not be over two millimeters in diameter—better, about one and one-half millimeters. It should be introduced into the fluid with the index finger pressed firmly over the top and lowered until the sediment is reached; then rotate the pipette, still keeping the finger pressed over the top, thus allowing the sediment to run in very slowly. By doing this we get as much sediment as possible in proportion to fluid, which is an important point when few casts are present. Before transferring to the slide, wipe off the fluid adhering to the outside of the pipette, otherwise that fluid containing no sediment will be deposited on the glass. It is better to use only a small drop on the slide and have only one layer of sediment to examine. It is easier, facilitates the rapid hunting over a slide and we are not so apt to miss delicate casts as if we were focussing rapidly through a long distance. Satisfactory work cannot be done with an amplification less than 350 diameters. Systematic examination of a slide is a material aid in hunting for casts. Beginning on one side go up and down on successive parallel lines until the whole slide has been traversed. Manipulation of the cover-glass with the fingers is apt to lead to error by rolling up mucus and granular matter, forming bodies not unlike casts in appearance.

If necessary there are many fluids in which an organised sediment may be preserved. The best is a solution of potassic acetate of specific gravity 1.050, to which has been added 95 per cent. carbolic acid in the proportion of 5 to 1,000. After settling and pouring off the supernatant urine add the preservative, let it settle and decant again. This process may be repeated two or three times to entirely replace the urine with preservative solution. The sediment may then be preserved in small bottles for future reference or mounted in shallow cells, the cover-glass being cemented down with one of the many cements used for this purpose. Bell's cement is as good, if not better, than any others. Asphaltum varnish should not be used, for in the course of a few years it cracks, allows the fluid to evaporate and thus spoil the mount.

325 Jersey Street.

---

A Liverpool physician makes a portable spirit lamp out of a thermometer case, by simply fitting it with a few strands of lamp cotton and then filling with spirits. Screw on the top and place a piece of rubber tubing over the joint, making it spirit tight. Good for sterilising needles. A suitable companion to Pavy's urinary test case and for other purposes.—*Atlanta Med. and Surg. Jour.*

